# Disruption of the Complex between GAPDH and Hsp70 Sensitizes C6 Glioblastoma Cells to Hypoxic Stress

**DOI:** 10.3390/ijms22041520

**Published:** 2021-02-03

**Authors:** Marina A. Mikeladze, Elizaveta A. Dutysheva, Victor G. Kartsev, Boris A. Margulis, Irina V. Guzhova, Vladimir F. Lazarev

**Affiliations:** 1 Institute of Cytology of the Russian Academy of Sciences, 194064 St. Petersburg, Russia; marinamikeladze.cytspb@gmail.com (M.A.M.); linza.uri@mail.ru (E.A.D.); margulis@incras.ru (B.A.M.); irina.guzh@gmail.com (I.V.G.); 2 InterBioscreen, 142432 Chernogolovka, Russia; vkartsev@ibscreen.chg.ru

**Keywords:** hypoxia, glioblastoma, glyceraldehyde-3-phosphate dehydrogenase, Hsp70, anticancer therapy, aggregates

## Abstract

Hypoxia, which commonly accompanies tumor growth, depending on its strength may cause the enhancement of tumorigenicity of cancer cells or their death. One of the proteins targeted by hypoxia is glyceraldehyde-3-phosphate dehydrogenase (GAPDH), and we demonstrated here that hypoxia mimicked by treating C6 rat glioblastoma cells with cobalt chloride caused an up-regulation of the enzyme expression, while further elevation of hypoxic stress caused the enzyme aggregation concomitantly with cell death. Reduction or elevation of GAPDH performed with the aid of specific shRNAs resulted in the augmentation of the tumorigenicity of C6 cells or their sensitization to hypoxic stress. Another hypoxia-regulated protein, Hsp70 chaperone, was shown to prevent the aggregation of oxidized GAPDH and to reduce hypoxia-mediated cell death. In order to release the enzyme molecules from the chaperone, we employed its inhibitor, derivative of colchicine. The compound was found to substantially increase aggregation of GAPDH and to sensitize C6 cells to hypoxia both in vitro and in animals bearing tumors with distinct levels of the enzyme expression. In conclusion, blocking the chaperonic activity of Hsp70 and its interaction with GAPDH may become a promising strategy to overcome tumor resistance to multiple environmental stresses and enhance existing therapeutic tools.

## 1. Introduction

Hypoxia is a phenomenon accompanying tumor growth and appears to be an essential factor for the progression of lung, colon, and brain cancers [1]. Hypoxia levels range from mild to severe, depending on the availability of blood vessels in adjacent healthy tissues [2]. Importantly, mild hypoxia can stimulate the growth and development of a tumor [3], while severe hypoxia causes serious disturbances in cellular physiology that may lead to cell death [4].

The response of tumor cells to hypoxia is regulated by a few transcription factors, causing significant changes in the homeostasis of cells and often leading to the adaptation of tumor cells to stress [5]. One such activator is hypoxia inducible factor alpha (HIF1α), which is known to trigger the transcription of genes involved in angiogenesis, erythropoiesis, proliferation, glycolysis, and other processes essential for survival of cancer cells [6].

The Warburg effect, known to boost the efficacy of glycolysis by several hundred times, serves as the adaptive mechanism for many tumors [7]. The crucial step of glycolysis is the conversion of glyceraldehyde-3-phosphate to 1,3-bisphosphoglycerate, which is catalyzed by glyceraldehyde-3-phosphate dehydrogenase (GAPDH); therefore, the amount of the active enzyme is of great importance for the energy metabolism of cancer cells under hypoxic conditions [8]. Hypoxia is also known to increase the number of reactive oxygen and nitrogen species caused by inactivation of the anti-oxidant system, leading to reoxygenation injury that causes chemical modifications of GAPDH, particularly oxidation and nitrosylation [9]. Such modifications lead to the inactivation of the enzyme and its transition to the aggregation-prone state [10]. Thus, the function of GAPDH in a tumor cell subjected to hypoxia is controversial; the enzyme is indispensable for cell survival due to promotion of the Warburg effect, whereas oxidative stress damages GAPDH molecules, targeting them to the nucleus and inducing apoptosis and/or converting them to an aggregated state that also results in cell death [11,12].

Another protein with a chaperonic activity and cytoprotective function associated with cell response to hypoxia and that plays a significant role in the protection of cancer cells is the Hsp70 chaperone (HSPA1A). Hsp70 prevents the formation of apoptosomes, binds the apoptosis-inducing factor AIF, and prevents apoptosis [13]. Hsp70 is a key element of the cellular system for recognizing and degrading proteins with disturbed conformation [14,15].

Interestingly, reduction of Hsp70 synthesis in C6 rat glioblastoma cells inhibited the chaperone’s effect on GAPDH aggregation and caused increased levels of cell death [16]. Since the expression of Hsp70 may be initiated by hypoxia, it seems reasonable that, in stressed cells, the chaperone functions in its conventional manner, recognizing damaged molecules of the enzyme and limiting their aggregation [17]. Therefore, in cancer cells, Hsp70-mediated chaperoning of GAPDH becomes a promising target for antitumor therapy. This approach implies a decrease in the stability of GAPDH–Hsp70 complex in hypoxic conditions due to impaired chaperoning of the enzyme attained with the aid of a special small molecule.

We previously showed that N-amino-ethylamino derivative of colchicine (AEAC) can bind Hsp70 and interfere with its protective function in rat C6 glioma cells and B16 mouse melanoma cells [18]. However, the exact mechanism of action of AEAC remained unclear—it was not known exactly which interaction of Hsp70 with client proteins was disrupted by AEAC. We hypothesized that AEAC is capable of inhibiting Hsp70-mediated chaperoning of damaged GAPDH during hypoxia. In the present work, we summarize the data obtained in studies of GAPDH function in hypoxia-imitating conditions and report the anti-tumor effect of AEAC, capable of dissociating GAPDH–Hsp70 interaction in cell and animal models of C6 rat glioblastoma.

## 2. Results

### 2.1. Pro-Tumor Effects of CoCl_2_ in Rat Glioma Cells

**Figure 1:**
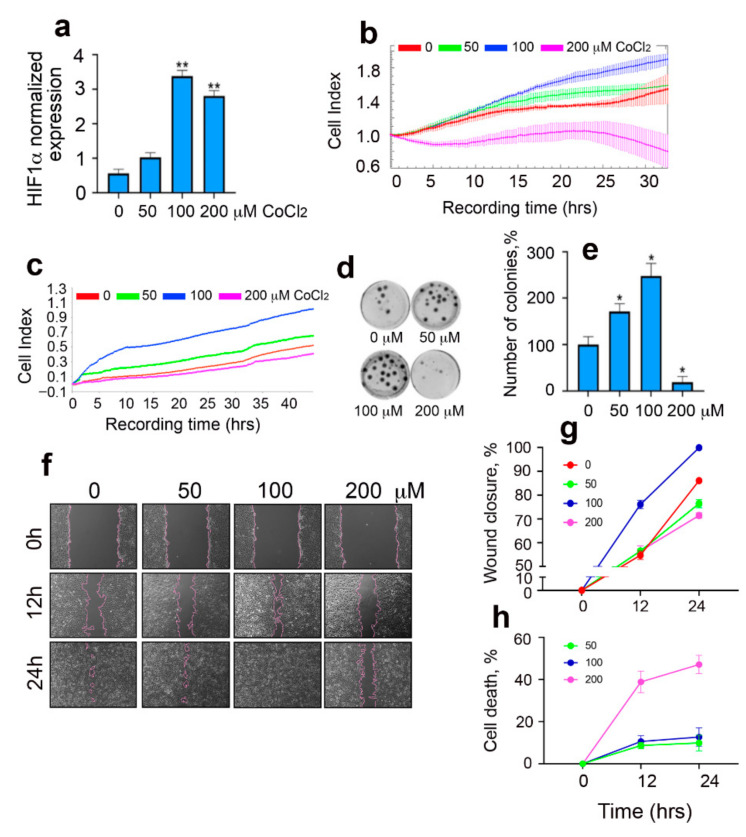
CoCl_2_ affects the proliferation, migration, and colony formation of rat glioma cells in a hypoxic model. (**a**) The data of RT-PCR are presented. Histogram bars show the relative amount of mRNA transcribed from the *hif1α* gene in C6 cells 6 h after addition of CoCl_2_; the data are normalized to the amount of actin mRNA. (**b**) Cell index data were obtained using the xCELLigence device. The graph shows the dynamics of the C6 cellular index placed in E-plates; CoCl_2_ was added in concentrations of 50, 100, and 200 μM after cell attachment to the bottom; incubation time is indicated on the *x*-axis. Representative data from three independent experiments are shown. (**c**) Data on C6 cell motility data obtained using the xCELLigence system. The graph shows the dynamics of the cellular index measured in the upper wells of the Cell Invasion and Migration (CIM) plates of the xCELLigence system in a medium containing 50, 100, or 200 μM CoCl_2_. The lower chambers of the CIM plates were filled with CoCl_2_-free medium. The recording of cell migration through the microporous membrane of the CIM plates lasted 40 h. Representative data from three independent experiments are shown. (**d**) C6 cell colony formation efficacy in the presence of 50, 100, and 200 μM CoCl_2_. (**e**) The result of colony number quantification from section (**c**) is presented. The histogram bars show the average normalized number of colonies based on three independent experiments. (**f**,**g**) Wound healing assay was performed with the aid of the JuLI Stage microscope and monitored by JuLI software. Cells were cultivated for 24 h in the presence of CoCl_2_ in different concentrations before the monolayer was scratched. The wound healing was detected with microscopy (**f**) and quantified with the aid of JuLI software (**g**). (**h**) The results of the CytoTox96 assay. C6 cells were incubated with CoCl_2_ for 24 h. Data presented as mean ± standard error of mean (SEM). Statistical significance is indicated as * *p* < 0.05 and ** *p* < 0.01.

We used the well-known HIF1α inducer cobalt chloride (CoCl_2_) to imitate hypoxia in glioma cells [19]. To confirm the adequacy of the hypoxic model at the molecular level, we analyzed the change in the level HIF1α expression in C6 cells after 24 h of incubation in the presence of CoCl_2_ with the aid of real time polymerase chain reaction (RT-PCR). As expected, the level of HIF1α mRNA increased 1.8-fold in the presence of 50 μM CoCl_2_, 6-fold in the presence of 100 μM CoCl_2_, and 5-fold in the presence of 200 μM CoCl_2_ (Figure 1a). Since the increase in HIF1α mRNA level was established to indicate the reduction of the oxygen concentration in the cell, which is parallel with the elevation of the cancer cell growth rate [20], we measured the latter value in a C6 cell population using the xCELLigence system (Figure 1b). We found that CoCl_2_ at concentrations of 50 and 100 μM caused an increase in the cell index, which indicated augmentation of cell growth. More severe hypoxia led to a reduction in the cell index, which was most probably due to elevated levels of cell death. To estimate another important feature of growing tumors, cell motility, we used special Cell Invasion and Migration (CIM) plates in the xCELLigence system and analyzed the impedance of cells crawled to the back of the inserts. We found that moderate hypoxia imitated by 50 and 100 μM CoCl_2_ led to an increase in cell motility by approximately 24% and 40%, respectively, compared to that in the control; higher doses of CoCl_2_ led to a substantial decrease in the cell index (Figure 1c). The ability of cells to form colonies was also analyzed as a part of the validation of the hypoxia model by CoCl_2_. The use of CoCl_2_ at concentrations of 50 and 100 μM increased the ability of C6 cells to form colonies by 71% ± 17% in the presence of 50 μM CoCl_2_ and by 148% ± 27% in the presence of 100 μM CoCl_2_ (Figure 1d,e). However, the increase in CoCl_2_ concentration up to 200 μM resulted in reduction of colony number by 80%, and at higher concentrations, colonies were not formed at all. Complementary results were also obtained using the scratch overgrowth test. The use of CoCl_2_ at a concentration of 50 μM did not cause significant changes; 100 μM CoCl_2_ accelerated the process of scratching, and at a concentration of 200 μM slowed the healing of scratches (Figure 1f,g). Next, we determined the cytotoxic effect of CoCl_2_ after 24 h of incubation with C6 cells using the CytoTox96 kit. Concentrations of 50 and 100 μM did not cause significant cell death, while culturing C6 cells in the presence of 200 μM CoCl_2_ for 24 h caused the death of 47% of the cell population (Figure 1h). These data demonstrated that the imitation of hypoxic stress gives a realistic pattern of effects observed in the cell population in hypoxic conditions and that moderate hypoxia leads to the activation of proliferation and increased motility of cancer cells, exerting a life-asserting effect on the latter.

### 2.2. Hypoxia Causes Increased Synthesis and Aggregation of GAPDH

One of the proteins synthesized under the control of HIF1α in cells responding to hypoxia should be GAPDH, which is necessary to maintain glycolysis in harmful conditions; hypoxia itself may also trigger the synthesis of stress proteins, including Hsp70. Therefore, at the next step, we studied the effect of CoCl_2_ on the amount of GAPDH and Hsp70 mRNA and proteins.

**Figure 2:**
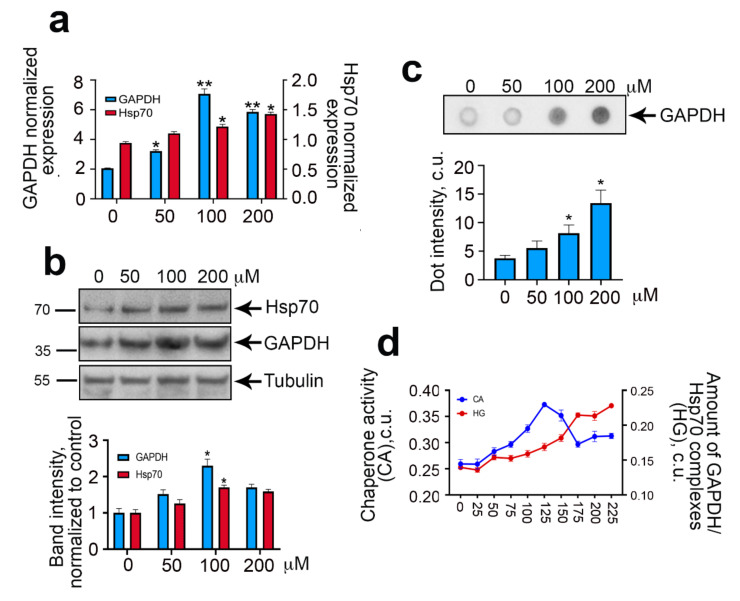
CoCl_2_ induces GAPDH expression and aggregation in C6 cells. (**a**) The data of RT-PCR are presented. Histogram bars show the relative normalized expression of *gapdh* and *hsp70* genes in C6 cells 6 h after addition of CoCl_2_. Actin was used as a reference gene. (**b**) Immunoblotting data are presented in the upper panel. C6 cells were treated with CoCl_2_ in the concentrations indicated. Cells were analyzed 24 h after addition of CoCl_2_. Tubulin is presented as a loading control. The data of immunoblotting quantification are presented in the lower panel. Histogram bars show the relative intensity of GAPDH and Hsp70 bands to intensity of tubulin bands normalized to this meaning for naïve cells. (**c**) C6 cells were incubated with CoCl_2_ for 24 h, then lysed and subjected to dot ultrafiltration, and the membrane was probed with anti-GAPDH antibodies (upper panel); dot intensity is presented in the lower panel. (**d**) C6 cells were incubated with CoCl_2_ for 24 h, then lysed. The Hsp70 chaperone activity and number of Hsp70–GAPDH complexes were measured in cell lysates with the aid of a protein–protein interaction assay. Data presented as mean ± standard error of mean (SEM). Statistical significance is indicated as * *p* < 0.05 and ** *p* < 0.01.

Using RT-PCR we demonstrated that incubation of C6 cells in the presence of 100 μM CoCl_2_ caused the elevation of *gapdh* gene transcription by 3.45 folds (Figure 2a). The increase in the expression of the *hsp70* gene was not so significant, since CoCl_2_ at a concentration of 100 μM caused a 1.3-fold increase in the mRNA. We also proved the accumulation of both proteins GAPDH and Hsp70 using Western blot analysis (Figure 2b). Treatment of cells with 100 μM CoCl_2_ for 24 h led to a 2.3-fold elevation of GAPDH content and 1.7-fold increase in Hsp70 amount (Figure 2b). Since hypoxia is accompanied by a burst of oxygen radicals and consequently with protein denaturation, we checked whether incubation with CoCl_2_ could affect GAPDH state. To estimate the number of aggregates, we employed a filter trap assay in which cell lysate in solution containing SDS was sucked through the acetate cellulose membrane in the 96-well manifold, and dots of proteins in aggregated form were stained with anti-GAPDH antibody (Figure 2c). As expected, the number of GAPDH-containing aggregates in cells became higher and elevated proportionally to the rising of the CoCl_2_ concentration. Moreover, we found significant differences from untreated cells, even at the lowest CoCl_2_ concentration of 50 μM. Thus, when simulating hypoxia using CoCl_2_ on C6 cells, we observed the activation of transcription of the *gapdh* gene, the accumulation of the enzyme, and the formation of GAPDH-containing aggregates.

To elucidate the role of Hsp70 in protecting cells from GAPDH aggregates under hypoxic conditions, we analyzed the chaperone activity of Hsp70 in the lysates of C6 cells treated with CoCl_2_ at various concentrations (Figure 2d). To measure the activity of Hsp70, we employed a substrate-binding assay in which Hsp70 was allowed to recognize denatured protein, carboxymethylated lactalbumin, or denatured GAPDH, as in this study. The amount of Hsp70 bound to the substrates was analyzed with the aid of specific antibodies [18]. We found that the increase in the concentration of CoCl_2_ caused an elevation of Hsp70–GAPDH complexes in cell lysates. At the same time, the chaperone activity of Hsp70 increased up to a concentration of 125 μM CoCl_2_ and then dropped down, suggesting that an increase in hypoxia severity caused the depletion of the active Hsp70 pool due to its interaction with GAPDH.

### 2.3. GAPDH–Hsp70 Interaction May Be Disrupted by AEAC Compound

Hypoxia mimicked here by the treatment with CoCl_2_ was found to elevate GAPDH content and probably its tumorigenic potential; the influence of the enzyme on cell homeostasis also extends to Hsp70, the total chaperonic capacity of which in a cell was partially deprived by GAPDH molecules. Binding of the enzyme to Hsp70 could be a factor supporting pro-tumor function of GAPDH and, more significantly, preventing the enzyme molecules from forming cytotoxic aggregates. Therefore, a substance capable of dissociating complexes of the two polypeptides can promote hypoxia-mediated denaturation of GAPDH and its subsequent aggregation.

**Figure 3:**
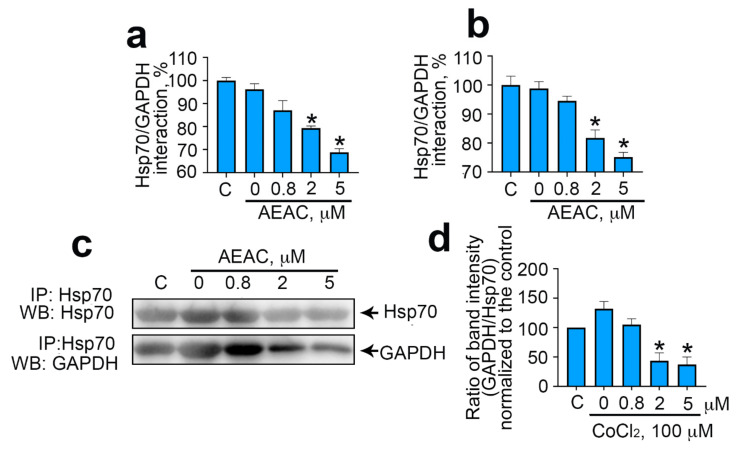
AEAC prevents interaction between GAPDH and Hsp70. (**a**) The data of the protein–protein interaction assay with pure Hsp70. GAPDH was immobilized on the bottom of a 96-well plate, then pure Hsp70 in the presence of different AEAC concentrations was added, and the chaperone was visualized with the aid of specific antibodies. Histogram bars show the normalized value of the GAPDH–Hsp70 interaction detected with the aid of 2H9 antibody. (**b**) The data of the protein–protein interaction assay with C6 cell lysate. GAPDH was immobilized on the bottom of the plate, then the lysate of pretreated AEAC C6 cells was added. Histogram bars show the normalized value of the GAPDH–Hsp70 interaction detected with the aid of 2H9 antibody. (**c**) Hsp70 was precipitated with the aid of 2H9 antibodies from lysates of cells treated with AEAC in concentrations as indicated in the figure. The immunoprecipitates were analyzed with the aid of Western blotting using antibodies with Hsp70 (upper panel) and GAPDH (lower panel). (**d**) The data of immunoblotting quantification. Histogram bars show the relative intensity of the GAPDH band to that of the Hsp70 band normalized to this value for naïve cells. Data presented as mean ± standard error of mean (SEM). Statistical significance is indicated as * *p* < 0.05.

To destroy the Hsp70–GAPDH complex, we used the AEAC compound, which previously demonstrated the ability to inhibit the chaperone activity of Hsp70 [18]. To assess the effectiveness of AEAC in inhibiting the interaction of purified Hsp70 and GAPDH, we used a protein–protein interaction assay (Figure 3a). We found that AEAC at concentrations from 0.8 to 5 μM interfered with the interaction of Hsp70 and GAPDH. Moreover, the use of the chemical at a maximal concentration reduced the efficiency of protein–protein interaction by 31.2% ± 1.6%. Similar results were obtained using a C6 cell lysate as a source of Hsp70 in a protein–protein interaction assay. The presence of AEAC decreased the binding efficiency of the immobilized on plate surface GAPDH and Hsp70 contained in the cell lysate. The inhibition efficiency depended on the concentration of AEAC and reduced by 24.9% ± 1.65% when using 5 μM AEAC (Figure 3b). In order to evaluate the effectiveness of disruption of the Hsp70–GAPDH complex, we used immunoprecipitation (Figure 3c). Thus, culturing C6 cells in the presence of AEAC led to the dissociation of the intracellular GAPDH–Hsp70 complex; for instance, the use of AEAC at a concentration of 5 μM caused a decrease in the number of the complexes by 78% ± 12.64% compared to cells treated with DMSO (Figure 3d).

### 2.4. The Role of Hsp70-Mediated Chaperoning of GAPDH in Cells Subjected to Hypoxia

**Figure 4:**
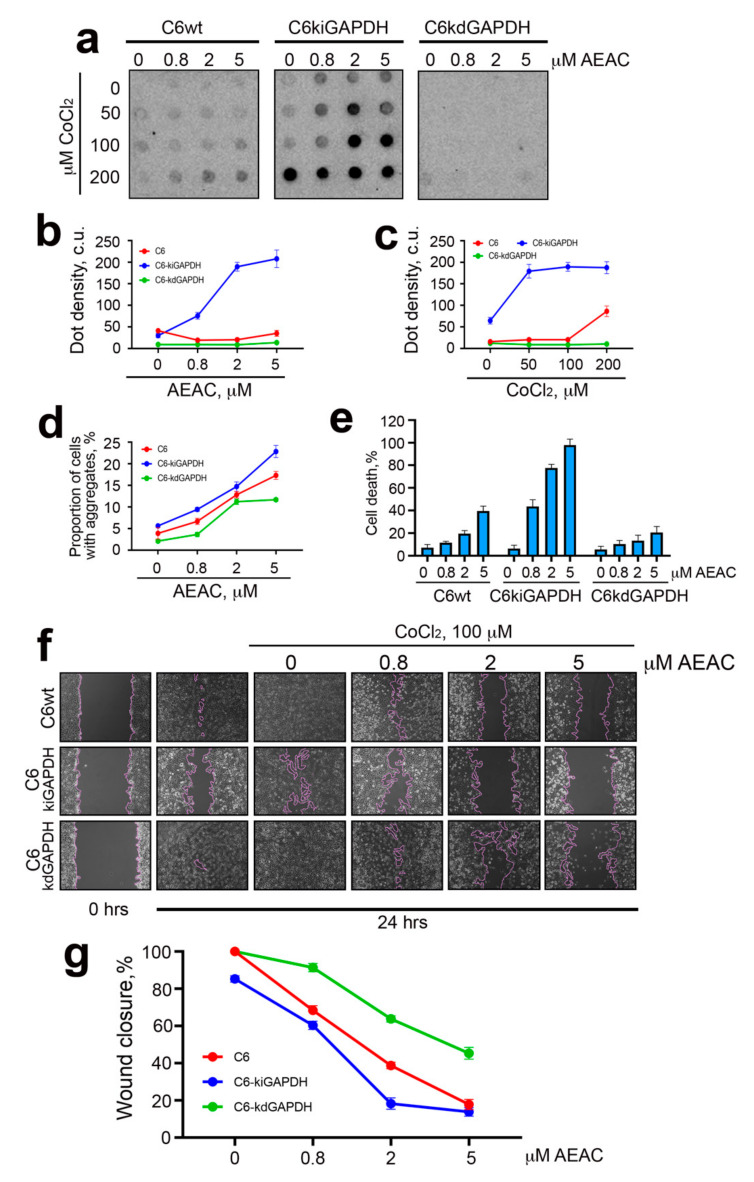
The level of GADPH expression affects the intensity of the enzyme aggregation, viability, and motility of cells during hypoxia. (**a**) C6 cells with various GAPDH levels were incubated with different concentrations of CoCl_2_ and AEAC for 24 h, then lysed and subjected to ultrafiltration. The membrane was probed with anti-GAPDH antibodies. (**b**,**c**) The data of dot intensity quantification are presented. The result for cells incubated in the presence of 100 µM CoCl_2_ and different AEAC concentrations is shown in (**b**); the result for cells incubated in the presence of 2 µM AEAC and different CoCl_2_ concentrations is shown in (**c**). (**d**) The proportion of cells with aggregates measured with the aid of a CX7 device is presented. (**e**) The results of the CytoTox96 assay. C6 cells were incubated with 100 µM CoCl_2_ and AEAC (0.8, 2, or 5 µM) for 24 h. (**f**,**g**) A wound healing assay was performed with the aid of a JuLI Stage microscope. Cells were cultivated for 24 h in the presence of 100 µM CoCl_2_ and AEAC (0.8, 2, or 5 µM) before the monolayer was scratched. The wound healing was detected with microscopy (**f**) and quantified by JuLI software (**g**).

Given the efficacy of AEAC in dissociating the Hsp70–GAPDH complex, we focused on the analysis of the compound effects on C6 cells subjected to treatment with CoCl_2_. In order to prove the critical role of GAPDH in the viability of tumor cells, we developed two cell lines on the base of parent C6 cells, C6-kiGAPDH (with enhanced GAPDH expression) and C6-kdGAPDH (with reduced GAPDH expression) using lentivirus constructs; the changes in GAPDH level in newly generated cells were proven with the aid of Western blotting (Figure S1). Then, we estimated the number of GAPDH-containing aggregates in cells expressing distinct levels of the enzyme and subjected to CoCl_2_; to estimate aggregation capacity, we employed the filter trap assay (Figure 4a). The data obtained from measurement of dot intensity showed that the treatment with CoCl_2_ induced the maximal degree of aggregation in C6-kiGAPDH cells, while in C6-kdGAPDH, GAPDH aggregation was almost undetectable, even after treatment with 100 μM CoCl_2_ (Figure 4a,b). Importantly, treatment with AEAC stimulated GAPDH aggregation, particularly in the enzyme overexpressing cells (Figure 4c).

Next, we analyzed the proportion of cells containing GAPDH aggregates using a CX7 device (Figure 4d). We found that the use of 5 μM AEAC combined with moderate hypoxia (100 μM CoCl_2_) caused an increase in the proportion of cells containing GAPDH aggregates, and the share of such cells was dependent on the level of GAPDH expression and comprised the following numbers: 17.3% ± 0.93% for C6, 22.8% ± 1.4% for C6-kiGAPDH, and 11.7% ± 0.36% for C6-kdGAPDH (Figure 4d). Confocal photographs of cells cultured with various concentrations of CoCl_2_ and AEAC are presented in Figure S2.

To substantiate the physiological significance of GAPDH aggregation in rat glioma cells, we used the CytoTox96 assay based on lactate dehydrogenase activity determination in cell medium. C6 cells with different GAPDH content were subjected to treatment with 100 μM CoCl_2_ (moderate hypoxia) in the presence of AEAC (Figure 4e). First, we showed that the use of AEAC caused elevation of cell death levels in conditions of hypoxia. In addition, we demonstrated that, in hypoxic conditions, the cells with the highest level of GAPDH expression were more sensitive to the action of AEAC, while cells with the lowest level of GAPDH expression showed the highest survival rate. Thus, after cultivation in the presence of 5 μM AEAC, the percentage of dead cells was 39.56% ± 4.29 for C6, 97.9% ± 5.22% for C6-kiGAPDH, and 20.55% ± 5.29% for C6-kdGAPDH (Figure 4e). Finally, we evaluated the growth dynamics of cells with different levels of GAPDH expression in the presence of CoCl_2_ (100 μM) and AEAC using a scratch overgrowth test (Figure 4f,g). The results of this experiment for 50 and 200 μM CoCl_2_ are presented in Figure S3. The cells overexpressing GAPDH endured hypoxia worse, and the effect of the use of AEAC in these cells was much more pronounced. The most interesting data were obtained in an experiment where the AEAC concentration was 2 μM. We showed that the effect of hypoxia and drug was strongly dependent on GAPDH content, so the scratch was most rapidly overgrown in a cell culture with a low GAPDH content (63.75% ± 2.88%), and most slowly in the culture with a high GAPDH content (18.24% ± 5.27%); in the control, for comparison, this parameter was 38.68% ± 3.06% (Figure 4f,g).

Such results clearly demonstrate that participation of GAPDH in aggregation processes can serve as a regulator of the viability of glioma cells under hypoxic conditions. It is important that the inhibition of Hsp70 chaperoning of GAPDH with the aid of AEAC reduced the resistance of glioma cells to moderate and severe hypoxia.

### 2.5. The Effect of GAPDH on the Glioma Tumor Progression in Animal Model

**Figure 5:**
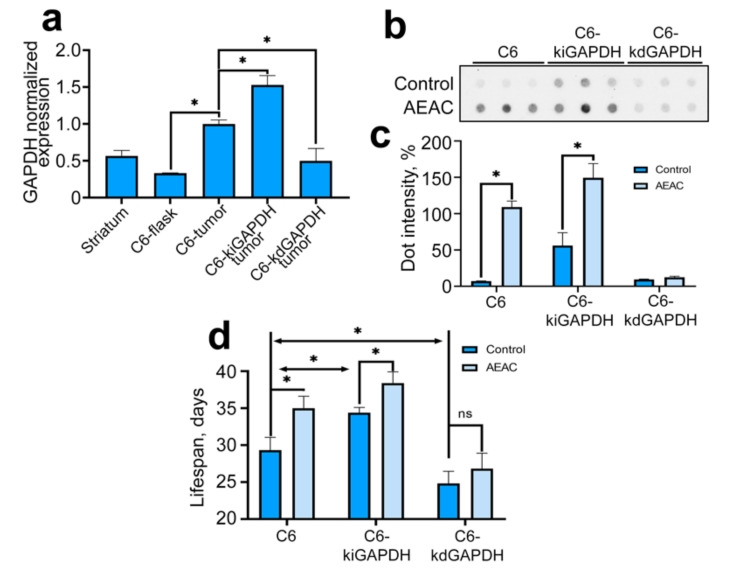
The level of GADPH expression in glioma tumors affects the enzyme aggregation and the lifespan of experimental rats. (**a**) The data of RT-PCR are presented. Histogram bars show the relative amount of mRNA transcribed from the *gapdh* gene in C6 cells cultivated in flasks and injected in rat brain normalized to the amount of actin mRNA. (**b**) Tumors formed in rats (treated or non-treated) from C6 cells with different GAPDH level were lysed and subjected to dot ultrafiltration. The resulting membrane was probed with antibody against GAPDH. Each dot illustrates the result for one rat’s tumor. (**c**) The average calculated dot intensity is presented. (**d**) The histogram bars illustrate the lifespan of rats after C6 cell injection. Data presented as mean ± standard error of mean (SEM). Statistical significance is indicated as * *p* < 0.05.

The physiological relevance of GAPDH content in glioma cells and the disruption of its chaperoning during hypoxia was studied in an in vivo glioblastoma model. We introduced C6 cells with different levels of GAPDH expression to the striatum area of rat brain. To verify hypoxic conditions in growth tumors, we analyzed the amount of HIF1α and GAPDH mRNA in glioma cells 21 days after their injection. We showed that the level of HIF1α mRNA in growing tumors was increased as compared to C6 cells cultivated in a flask (Figure S4a), which indicated the cell response to hypoxia. GAPDH mRNA levels were also increased in tumor cells compared to those in C6 cells cultured in flask, so in C6 cells in the tumor, the level of GAPDH was increased 3-fold; in C6-kiGAPDH cells, 4.6-fold; and in C6-kdGAPDH cells it did not alter significantly from the level of GAPDH expression in cultured C6 cells but was 2-fold less than that in C6 cells of the tumor (Figure 5a and Figure S4b). Next, we analyzed the number of GAPDH-containing aggregates in tumors at 21 days after injection of C6 cells with different levels of GAPDH expression during therapy with AEAC (Figure 5b,c). We found that the number of GAPDH aggregates was greater in tumors formed by cells with elevated GAPDH compared to that in tumors with normal GAPDH level. The level of GAPDH aggregation in cells with reduced and normal GAPDH expression did not differ. Therapy with 2 mg/kg AEAC significantly increased the number of GAPDH-containing aggregates in tumors formed by cells with normal and increased levels of GAPDH expression. Finally, we estimated the life expectancy of animals with tumors formed by cells with distinct GAPDH content (Figure 5d). First, we found that the lifespan of animals with tumors formed by cells overexpressing GAPDH was longer than the lifespan of animals with tumors formed by cells with a normal GAPDH level: 34.4 days and 29.3 days, respectively.

Secondly, we demonstrated that AEAC increased the life expectancy of animals with tumors formed by C6 cells (by 19.5%) and C6-kiGAPDH (by 11.6%) and did not affect the life expectancy of animals with tumors formed by C6-kdGAPDH cells (Figure 5d). These data indicate that under conditions of hypoxia due to a lack of oxygen, GAPDH is inclined to form cytotoxic aggregates that inhibit further tumor growth, and the AEAC compound, by disrupting the Hsp70-mediated chaperoning of GAPDH, controls this process.

## 3. Discussion

One of the key regulators of cell response to hypoxia is HIF1α, the activation of which leads to an increase in VEGF expression and through the VEGF-2 receptor to the enhancement of cancer cell growth and migration [21]. We managed to select CoCl_2_ concentrations at which these obvious manifestations of hypoxia in rat C6 glioma cells were observed and found that the incubation of C6 cells with 100 μM CoCl_2_ caused an increase of GAPDH expression and an almost 2.2-fold elevation of the enzyme content. It is known that overexpression of GAPDH usually leads to increased enzyme aggregation and to the reduced viability of cancer cells in the models of rotenone- [22] and nitric oxide-induced [23] cell death. Oxidative stress occurring apparently as a result of mitochondria dysfunction and usually accompanying hypoxia may also cause the oxidation of the enzyme and formation of its cytotoxic aggregates as was earlier found in quite similar experiments [24]. The relevance of GAPDH content for cell viability in hypoxic conditions was convincingly proved in experiments where we analyzed the response of C6 cells expressing distinct levels of the enzyme to CoCl_2_. Most remarkable was the fact that knockdown of GADPH resulted in minimal cell death, even at the highest concentration of CoCl_2_; vice versa, cells with up-regulated GAPDH levels were maximally susceptible to hypoxia imitation (Figure 4e). Importantly, these indications of GAPDH relevance for a tumor cell fate were obtained both in cell and animal models.

Thus, two consequences of hypoxia are likely to be deleterious for a cancer cell experiencing hypoxia, reduced efficiency of GAPDH in glycolysis and the Warburg effect [11,25] and its aggregation due to increased expression of the enzyme and its denaturation [16].

Proteotoxic insults are usually managed by molecular chaperones, typically Hsp70, which was earlier demonstrated to bind GAPDH and reduce its aggregation capacity. In this study, we showed that, at a certain degree of hypoxic stress, the cells synthesize faintly increased amounts of Hsp70, and most importantly, exhibited elevated total chaperonic capacity as proved by the data of a special immune-enzyme assay (Figure 2d). Of note, the elevation of the CoCl_2_ level to 125 mM led to further growth of the number of GAPDH–Hsp70 complexes corroborated with the reduction of chaperone activity. We suggest that the elevated number of GAPDH aggregates occurring at a CoCl_2_ concentration above 100 μM caused the excessive occupancy of the chaperone, or in other words, the reduction of its activity. Thus, an important result of our study is a demonstration of the effect of GAPDH level on cell viability under hypoxic conditions. In both in vitro and in vivo models, we showed that increased GAPDH levels reduce the resistance of tumor cells to hypoxia.

Hsp70 has plenty of resources to support cell viability; the chaperone can interfere with major pathways of apoptosis, affect particular proteolytic mechanisms to make them more efficient, and inhibit the activity of major transcription factors, such as NF-kappaB and many others [26,27]. Earlier processing of caspase 3 and 7 was found to be inhibited due to their trapping by the chaperone [28]. In this study, we found that Hsp70 may support a pool of GAPDH molecules in a latent form very similarly to that demonstrated for Hsp90 and its multiple client proteins [29]. Hypothetically, GAPDH (just by its quantitative supremacy) can substitute numerous targets of Hsp70, including those participating in apoptosis signaling.

Complexes between Hsp70 and other potential client proteins can be dissociated by the specific chaperone inhibitors; during last two decades, few of such compounds have been offered, including VER-155008 [30], JG-98 [31], MKT-077 [32], iCit-2 [33], and others. In this study, the colchicine derivative AEAC demonstrated high capacity for dissociation of the oxidized GAPDH from the Hsp70 chaperone, and the dissociation occurred irrespective of whether the initial complex was formed ex vivo or in vitro (Figure 3).

It is of importance that the AEAC-mediated release of GAPDH from its complex with Hsp70 and its anti-tumor action depended on GAPDH level. A high level of GAPDH correlated with the increased efficiency of AEAC in stimulation of GAPDH aggregation and prolonged survival of rats with tumors; vice versa, we did not detect significant effects of AEAC on tumor cells with low GAPDH level and the lifespan of animals that were injected with C6-kdGAPDH cells. We believe that the application of AEAC in glioma therapy may be effective due to its dual cytotoxic action: (1) disruption of GAPDH chaperoning, as indicated above, and (2) blocking of the antiapoptotic activity of Hsp70, as we showed earlier [18].

## 4. Materials and Methods

### 4.1. Cells and Chemicals

Rat glioma C6 cells and human embryonic kidney HEK-293T cells were obtained from the Russian Cell Culture Collection at the Institute of Cytology of the Russian Academy of Sciences (St. Petersburg, Russia). C6 cells were grown in Dulbecco’s modified Eagle’s medium/F12 medium supplemented with 10% fetal bovine serum (GE Healthcare, Chicago, IL, USA), 2 mM L-glutamine, and antibiotics (100 U/mL penicillin G and 0.1 mg/mL streptomycin). HEK-293T cells were grown in Dulbecco’s modified Eagle’s medium supplemented with 10% fetal bovine serum (GE Healthcare, Chicago, IL, USA), 2 mM L-glutamine, and the same antibiotics. Cells were grown in a CO_2_ incubator with 5% CO_2_ and 90% humidity at 37 °C. To imitate hypoxic conditions in C6 cells, we used cobalt chloride (CoCl_2_, Sigma-Aldrich, St. Louis, MO, USA) in concentrations from 50 to 200 µM.

To vary the GAPDH level in C6 cells, plasmids bearing the entire *gapdh* gene, pLOC-GAPDH (GAPDH knock-in, C6-kiGAPDH), and clone TRCN0000041460, shRNA to GAPDH (GAPDH knock-down, C6-kdGAPDH), mature antisense: CTGAGTATGTCGTGGAGTCTA were purchased from Thermo Fisher Scientific (Waltham, MA, USA). Packaging (Δ8.91) and envelope (pCMV-VSV-G) plasmids were purchased from Invitrogen (Carlsbad, CA, USA). The HEK-293T host cells were transfected using polyethyleneimine (Sigma-Aldrich, St. Louis, MO, USA) mixed with all three plasmids.

(S)-N-(10-((2-aminoethyl)amino)-1,2,3-trimethoxy-9-oxo-5,6,7,9-tetrahydrobenzo [a]heptalen-7-yl acetamide (AEAC) was obtained from InterBioScreen, Chernogolovka, Russia.

### 4.2. Proliferation and Migration Assays

The xCELLigence system (ACEA Biosciences, San Diego, CA, USA) provides non-invasive and label-free monitoring of cell viability, growth, and migration in real-time based on measurement of the electrical impedance of cells adhered to an electrode on the well bottom. Elevated impedance indicates that an increased number of cells adhered to the bottom at that moment compared to the previous time point. C6 cells were placed in 16-well E-plates (4.0 × 104 cells/mL; ACEA Biosciences, San Diego, CA, USA). After 12 h, C6 cells were cultivated with CoCl_2_ in the presence of AEAC. Cell proliferation was then monitored for 48 h using the RTCA xCELLigence System. To estimate the migration capacity, we employed CIM plates according to the instructions of the manufacturer. Data analysis was performed using RTCA Analysis Software (RTCA Software Pro, xCELLigence Instruments, ACEA, San Diego, CA, USA).

### 4.3. Colony Formation Assay

C6 cells were treated with CoCl_2_ in concentrations of 50, 100, and 200 µM for 24 h; washed; plated in six-well plates at a concentration of 100 cells/mL; and incubated for 9 days in 5% CO_2_ at 37 °C. Then, cells were fixed with 10% formaldehyde, stained with 0.01% crystal violet and dried. Plates were scanned with the aid of the ChemiDoc system (Bio-Rad, Hercules, CA, USA).

### 4.4. Wound Healing Assay

C6, C6-kiGAPDH, and C6-kdGAPDH cells were incubated in growth medium for 24 h. The C6 monolayer was wounded by scratching with a 5 mL pipette tip. After this, cells were incubated with CoCl_2_ in concentrations of 50, 100, and 200 µM and AEAC in concentrations of 0.8, 2, and 5 µM. Wound healing was monitored for 18 h with the aid of the JuLI Stage microscope (NanoEnTek, Seoul, Korea) and monitored by JuLI software V2.0.

### 4.5. RNA Isolation and Real-Time PCR

RNA was isolated using TRIzol (Thermo Fisher Scientific, Waltham, MA, USA) and reverse transcribed using the MMLV RT kit (Evrogen JSC, Moscow, Russia) according to manufacturer’s instructions. All RT-PCR reactions were performed with a CFX96 Real-Time PCR detection system (Bio-Rad, Hercules, CA, USA) using qPCRmix-HS SYBR (Evrogen JSC, Moscow Russia) according to the manufacturer’s protocol. Amplicon authenticity was confirmed by melt curve analysis. The sequence of primers was as follows for GAPDH: (forward) 5′-ATGATTCTACCCACGGCAAG-3′, (reverse) 5′-CTGGAAGATGGTGATGGGTT-3′; for HIF1α: (forward) 5′-GGTGGATATGTCTGGGTTGAG-3′, (reverse) 5′-TTCAACTGGTTTGAGGACAGA-3′; Hsp70: (forward) 5′-CTCGAGTCCTATGCCTTCAACA-3′, (reverse) 5′-CACTTGTCCAGCACCTTCTTCT-3′; actin: (forward) 5′-TATGTTGCCCTAGACTTCGAGC-3′, (reverse) 5′-CGATAGTGATGACCTGACCGTC-3′. Beta-actin was used as the normalization control. All primers were obtained from Evrogen JSC (Moscow, Russia). The parameters of the polymerase chain reaction (PCR) were 5 min of pre-denaturation at 95 °C, followed by 40 cycles of 30 s at 95 °C, 30 s at 65 °C, and 30 s at 70 °C. The data were analyzed for fold change (ΔΔCt) using Bio-Rad CFX software (version 3.1).

### 4.6. Analysis of Aggregates

Determination of GAPDH-containing aggregates was carried out using 2 tests: filter trap assay and quantification of aggregate amount with the aid of CellInsight CX7 High-Content Screening Platform (Thermo Fisher Scientific, Waltham, MA, USA).

For the filter trap assay, C6, C6-kiGAPDH, C6-kdGAPDH cells were incubated with CoCl_2_ in concentrations of 50, 100, and 200 µM and AEAC in concentrations of 0.8, 2, and 5 µM for 48 h. Cell lysates were filtered through a 0.22 mm cellulose acetate membrane using a 96-well TransBlot manifold (Bio-Rad, Hercules, CA, USA) in buffer containing 10 mM Tris HCl pH 8.0, 150 mM NaCl, and 2% sodium dodecyl sulphate (SDS). After filtration was completed, the cellulose acetate membrane was incubated in PBS containing 5% fat-free milk and probed with the 6C5 antibody against GAPDH (Abcam, Cambridge UK). Quantification of dot intensity was obtained with TotalLabQuant CLIQS V1 software.

For aggregate quantification, C6, C6-kiGAPDH, and C6-kdGAPDH cells were cultured in the same conditions as the filter trap assay in 96-well plates for 48 h. Then, cells were fixed in 4% paraformaldehyde (Sigma-Aldrich, St. Louis, MO, USA), incubated with 6C5 antibodies, and subsequently tagged with Alexa-fluor 488 conjugated goat anti-mouse antibodies (Abcam, Cambridge, UK). For cell nucleus visualization, we used 4′,6-Diamidino-2-Phenylindole, Dihydrochloride (DAPI, Thermo Fisher Scientific, Waltham, MA, USA). The aggregate quantification was carried out with the aid of the CX7 device with preset software.

### 4.7. Protein–Protein Interaction Assay

All procedures were performed at 37 °C, unless otherwise indicated. Oxidized GAPDH (3 mM H_2_O_2_ for 30 min) from rabbit muscle was diluted to a concentration of 5 mg/mL in PBS and immobilized for 1 h on a F96 MicroWell plate (Thermo Fisher Scientific, Waltham, MA, USA). The plate was washed with PBS, and non-specific binding was blocked with 5 mg/mL bovine serum albumin solution in PBS. Then, the immobilized GAPDH was allowed to react with purified Hsp70 or cellular extracts containing Hsp70 in the presence of AEAC in concentrations of 0.8, 2, and 5 µM for 2 h in a solution containing 25 mM Tris HCl pH 7.5, 20 mM NaCl, and 10 mM MgCl_2_. The amount of Hsp70 bound to the immobilized oxidized GAPDH was measured using 2H9 anti-Hsp70 monoclonal antibody [34], followed by anti-mouse IgG conjugated with peroxidase (Jackson ImmunoResearch, Cambridge, UK). Visualization was performed using the tetramethylbenzidine-containing reagent SUBS TMB (XEMA, St. Petersburg, Russia) according to manufacturer’s protocol. The colored staining was measured using a Charity Fluorofot reader (Probanauchpribor, St. Petersburg, Russia).

The effects of CoCl_2_ substrate-binding Hsp70 activity were explored, as described by [35]. Briefly, carboxymethylated lactalbumin (CMLA) 10 μg/mL in phosphate-buffered saline (PBS) was applied to the surface of a 96-well microplate. Non-specific binding was then eliminated by applying 5 mg/mL bovine serum albumin in PBS. The extracts were obtained from C6 cells that were incubated with CoCl_2_. The lysate of C6 cells in buffer containing 25 mM Tris HCl pH 7.5, 20 mM NaCl, and 10 mM MgCl_2_ were applied to wells. Next, 2H9 antibody was added, followed by anti-mouse IgG conjugated with peroxidase. Visualization was performed as described previously.

Determination of the number of GAPDH–Hsp70 complexes in cell lysates was also performed using a protein–protein interaction assay. For this purpose, rat polyclonal antibodies against GAPDH (obtained in our laboratory) at a concentration of 2 µg/mL were immobilized on the bottom of a 96-well plate. After blocking of non-specific binding, lysates of C6 cells pretreated with CoCl_2_ (as described above) were incubated in wells for 2 h at 37 °C. To determine the number of bound complexes, 2H9 antibodies followed by anti-mouse IgG conjugated with peroxidase were added to the wells. Visualization was performed as described previously.

### 4.8. Immunoprecipitation

C6 cells were incubated with 100 µM CoCl_2_ in the presence of AEAC in concentrations of 0.8, 2, and 5 µM for 24 h and lysed in a buffer containing 20 mM HEPES (pH 7.5), 100 mM NaCl, 1.5 mM MgCl_2_, 0.5 mM EDTA, 0.5 mM DTT, and 0.025% NP-40. After, lysate containing 0.5 mg total protein was incubated for 18 h with anti-Hsp70 antibody bound to Protein G-Sepharose (Sigma-Aldrich, St. Louis, MO, USA). The beads were washed in the above buffer with 0.05% Tween 20 and boiled in SDS-containing sample buffer and subjected to electrophoresis and Western blotting with the use of anti-GAPDH and anti-Hsp70 antibodies. The quantification of the interaction between GAPDH and Hsp70 was carried out as follows. Using TotalLabQuant software, we obtained the band intensity value in conventional units, then normalized the values for the Hsp70 bands to the appropriate value for naïve cells. Then a similar procedure was carried out for the GAPDH bands. Finally, the normalized value of the intensity of the GAPDH bands was divided by the normalized value of the intensity of the Hsp70 bands.

### 4.9. Animals

Male Wistar rats were purchased from an animal nursery (Rappolovo, St. Petersburg, Russia). Rats were anesthetized with 10 mg Zoletyl-100 (tiletamine hydrochloride and zolazepam, Carros CEDEX, France) and 0.2 mL 2% Rometar (xylazinum hydrochloride, Bioveta, Ivanovice na Hané, Czech Republic) injected intraperitoneally before being mounted in a stereotactic frame (SR-5R, Narishige Scientific Instrument Laboratory, Tokyo, Japan). C6, C6-kiGAPDH or C6-kdGAPDH cells were injected in the area of the striatum with the following coordinates: AP = 1; L = 2.5; DV = 5. Beginning on day seven after tumor cell inoculation, the groups were treated in different ways—group one was treated with DMSO (Control) and group two (AEAC) was treated with 2 mg/kg AEAC. AEAC was administered intraperitoneally, and treatment lasted 27 days (one application every two days). Each group contained 10 animals. Twenty days after tumor inoculation, 3 rats from each group were decapitated, and tumor tissues were analyzed with RT-PCR and the filter trap assay. For the remaining 7 animals, lifespan was recorded from the moment of tumor cell introduction.

All animal experiments were carried out in accordance with the guidelines for the welfare of animals of the Institute of Cytology, Russian Academy of Sciences No. F18-00380 (approved on 12 October 2017).

### 4.10. Statistics

All data were expressed as the mean ± standard error of the mean (SEM). Statistics were compared using the Mann–Whitney non-parametric test with the aid of GraphPad Prism 8 software. All experiments, excluding animal studies, were repeated at least three times. Statistical significance was determined by a value of *p* < 0.05.

## 5. Conclusions

In hypoxic conditions, a disruption of the Hsp70-mediated GAPDH chaperoning led to subsequent denaturation and aggregation of the enzyme that may cause the death of tumor cells. One of the ways to disrupt chaperoning of GAPDH may be the application of the Hsp70 binder, AEAC. We believe that this approach can be effective in the treatment of solid tumors experiencing hypoxia and at the same time containing high levels of GAPDH.

## Data Availability

Not aplicable.

## Supplementary Materials

The following are available online at https://www.mdpi.com/1422-0067/22/4/1520/s1, Figure S1: Verification of GAPDH expression level in C6-kiGAPDH and C6-kdGAPDH cell sublines; Figure S2: Fluorescent confocal images of C6 cells with different GAPDH level cultured with various concentrations of CoCl2 and AEAC; Figure S3: Analysis of the proliferation of C6 cells with different levels of GAPDH expression in the presence of AEAC under hypoxic conditions; Figure S4: Analysis of the expression of hif1α and gapdh genes in C6 cells with different GAPDH level in a flask and in rat brain.
