# Supplementary material for: Disruption of the Complex between GAPDH and Hsp70 Sensitizes C6 Glioblastoma Cells to Hypoxic Stress
Source: Int J Mol Sci. 2021 Feb 3;22(4):1520. doi: 10.3390/ijms22041520 (PMC7913589; doi:10.3390/ijms22041520)
Supplement: Supplementary file 1 [file ijms-22-01520-s001.pdf]

SUPPLEMENTARY INFORMATION

Disruption of the complex between GAPDH and Hsp70 sensitizes C6 glioblastoma cells to hypoxic stress

Marina A. Mikeladze [1], Elizaveta A. Dutysheva [1], Victor G. Kartsev [2], Boris A. Margulis [1], Irina V. Guzhova [1] and Vladimir F. Lazarev [1,*]

[1] Institute of Cytology of the Russian Academy of Sciences, 194064 St. Petersburg, Russia; marinamikeladze.cytspb@gmail.com (M.A.M.); linza.uri@mail.ru (E.A.D.); margulis@incras.ru (B.A.M.); irina.guzh@gmail.com (I.V.G.); vl.lazarev@gmail.com (V.F.L)

[2] InterBioscreen, 142432 Chernogolovka, Russia; vkartsev@ibscreen.chg.ru (V.G.K.)

* Correspondence: vl.lazarev@gmail.com; Tel.: +7-931-233-1811 (V.F.L.)

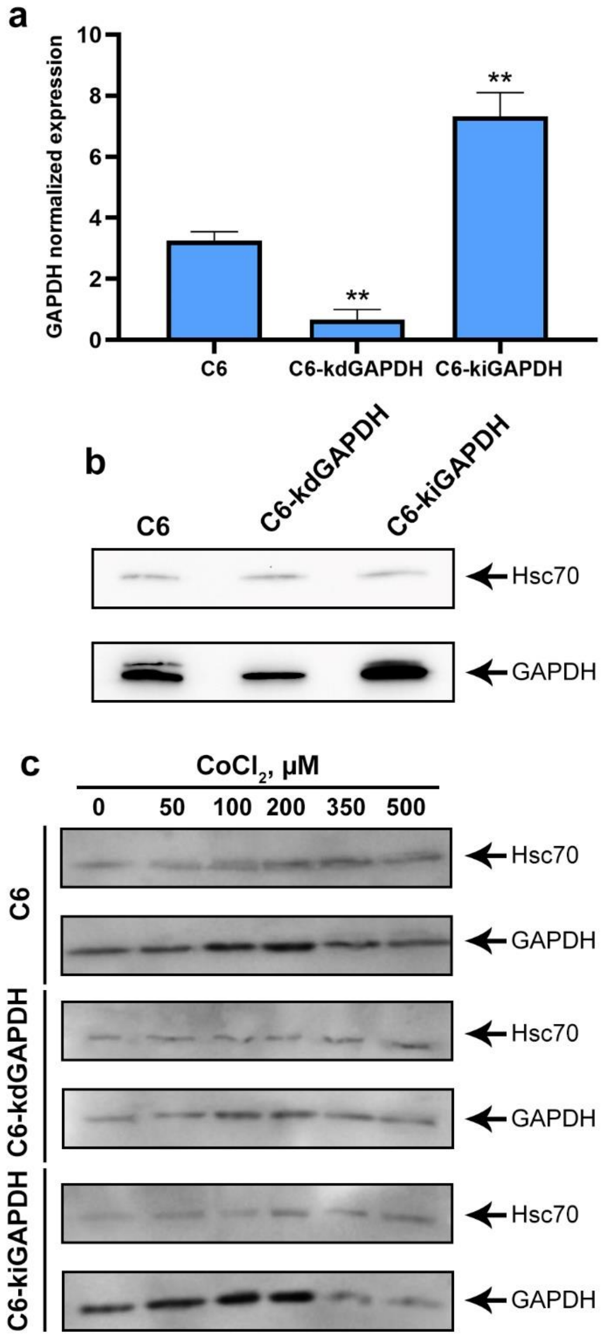

Figure S1. Verification of GAPDH expression level in C6-kiGAPDH and C6-kdGAPDH cell sublines. (**a**)The data of RT-PCR are presented. Histogram bars show the relative amount of mRNA transcribed from the *gapdh* gene in C6 cells of different sublines. (**b**) Immunoblotting data are presented. C6 cells of different sublines were lysed and analyzed with electrophoresis with subsequent Western blot staining. Antibodies against GAPDH were used to analyze the protein level in different cells. Hsc70 is presented as a loading control. (**c**) Immunoblotting data are presented. C6, C6-kdGAPDH and C6-kiGAPDH cells were treated with $CoCl_2$ in concentrations marked on the figure. Cells were analyzed 24 h after the addition of $CoCl_2$. Hsc70 is presented as a loading control.

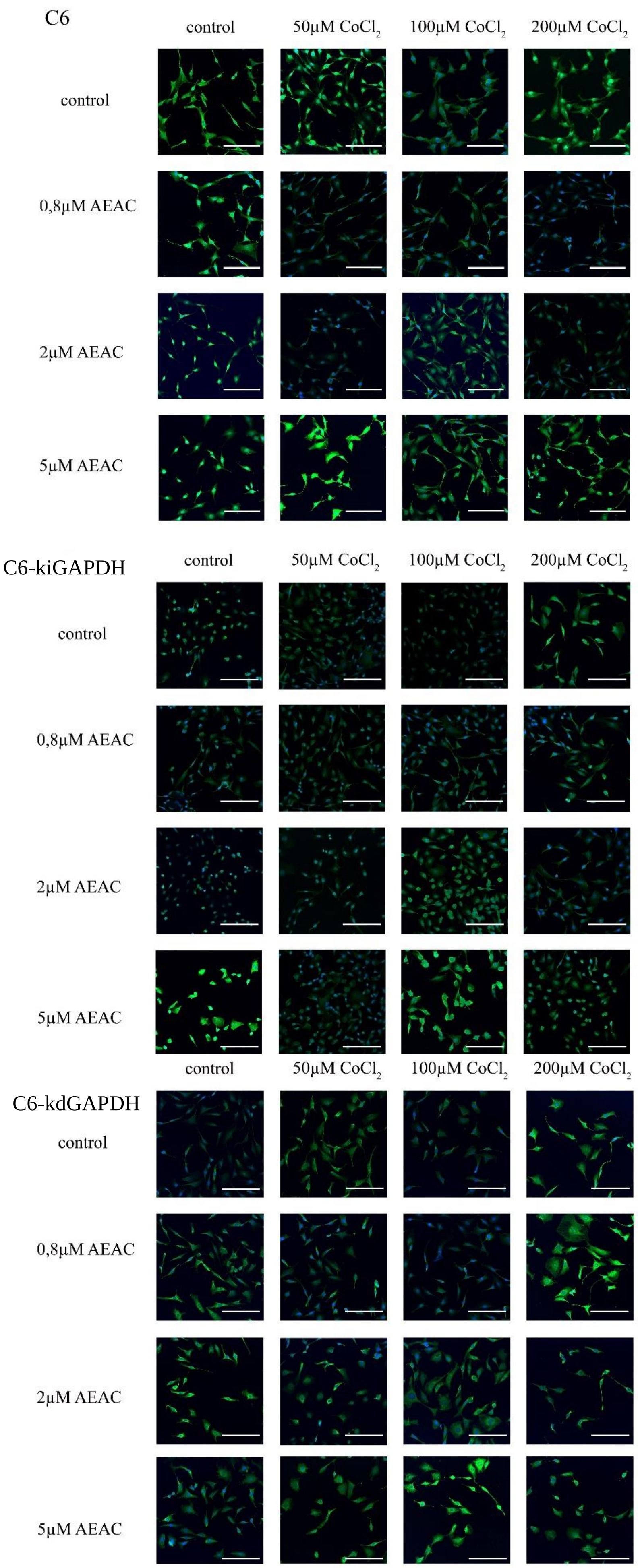

Figure S2. Fluorescent confocal images of C6 cells with different GAPDH level cultured with various concentrations of CoCl2 and AEAC. C6 (upper panel), C6-kiGAPDH (middle panel) or C6-kdGAPDH (lower panel) cells were cultivated for 24 h in the presence of $CoCl_2$ and AEAC in indicated concentrations. DAPI was used for nucleus labeling. Anti-GAPDH antibodies was labeled with Alexa-488. Scale bar 50 μm

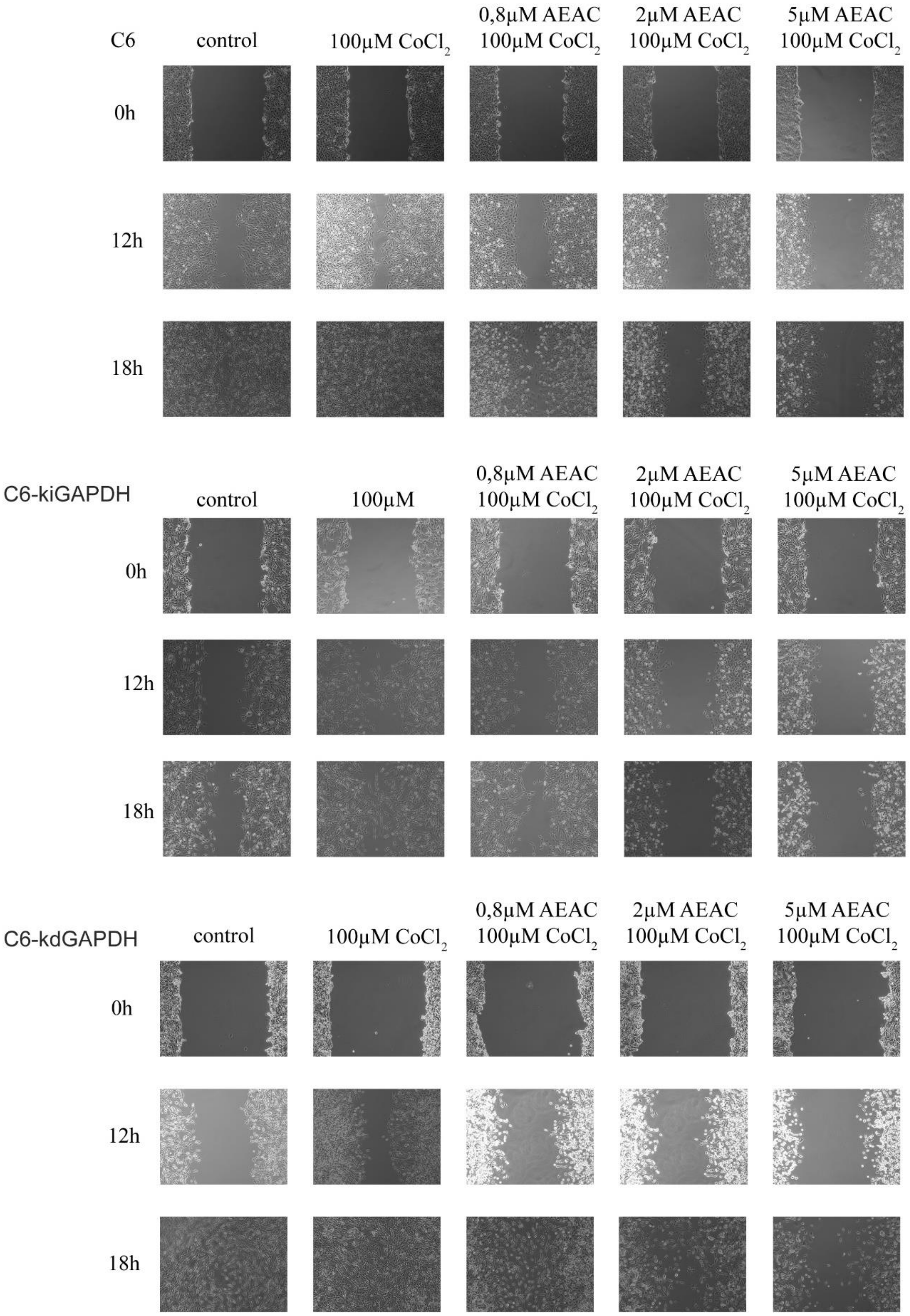

Figure S3. Analysis of the motility of C6 cells with different levels of GAPDH expression in the presence of AEAC under hypoxic conditions. Wound healing assay was performed with the aid of the JuLI Stage microscope. C6 (upper panel), C6-kiGAPDH (middle panel) or C6-kdGAPDH (lower panel) cells were cultivated for 24 h in the presence of 100 µM $CoCl_2$ and AEAC (0.8, 2 or 5 µM) before the monolayer was scratched. Wound healing was detected with microscopy.

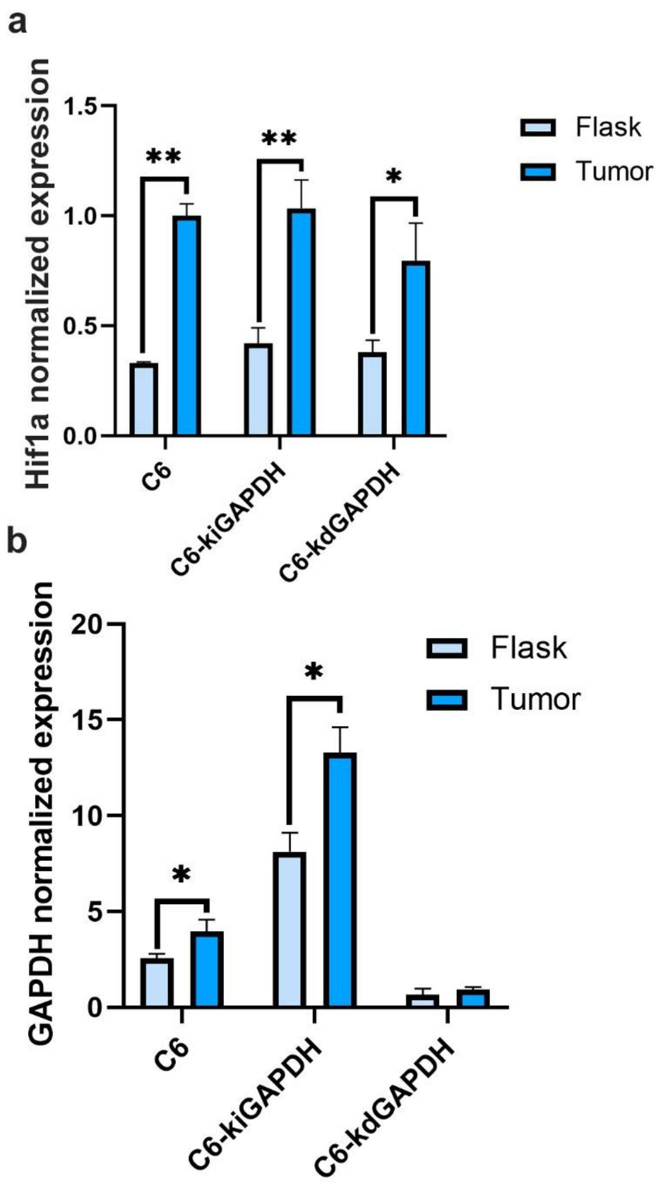

Figure S4. Analysis of the expression of *hif1α* and *gapdh* genes in C6 cells with different GAPDH level in a flask and in rat's brain. The data of RT-PCR are presented. Histogram bars show the relative amount of mRNA transcribed from the HIF1α (**a**) and *gapdh* (**b**) genes in C6, C6-kiGAPDH or C6-kdGAPDH cells cultivated in flasks and injected in rat's brain normalized to the amount of actin mRNA.
